# The interplay between neuronal activity and actin dynamics mimic the setting of an LTD synaptic tag

**DOI:** 10.1038/srep33685

**Published:** 2016-09-21

**Authors:** Eszter C. Szabó, Rita Manguinhas, Rosalina Fonseca

**Affiliations:** 1 Cellular and Systems Neurobiology, Instituto Gulbenkian de Ciência, Rua da Quinta Grande, 6, 2780-156 Oeiras Portugal

## Abstract

Persistent forms of plasticity, such as long-term depression (LTD), are dependent on the interplay between activity-dependent synaptic tags and the capture of plasticity-related proteins. We propose that the synaptic tag represents a structural alteration that turns synapses permissive to change. We found that modulation of actin dynamics has different roles in the induction and maintenance of LTD. Inhibition of either actin depolymerisation or polymerization blocks LTD induction whereas only the inhibition of actin depolymerisation blocks LTD maintenance. Interestingly, we found that actin depolymerisation and CaMKII activation are involved in LTD synaptic-tagging and capture. Moreover, inhibition of actin polymerisation mimics the setting of a synaptic tag, in an activity-dependent manner, allowing the expression of LTD in non-stimulated synapses. Suspending synaptic activation also restricts the time window of synaptic capture, which can be restored by inhibiting actin polymerization. Our results support our hypothesis that modulation of the actin cytoskeleton provides an input-specific signal for synaptic protein capture.

Activity-dependent plasticity of synaptic connections reflects a combination of functional and structural alterations at synapses and represents a cellular model of memory formation^1^,^2^,^3^. Classic work on synaptic plasticity has shown that persistent forms of LTP and LTD require *de novo* protein synthesis of plasticity-related proteins (PRPs)^4^,^5^. Since LTP and LTD are by large input-specific it was proposed that activated synapses are tagged so that synthesised PRPs are captured at modified synapses (synaptic tagging and capture hypothesis - STC)^6^,^7^. The STC provides a cellular mechanism to associate transient and persistent forms of plasticity through the sharing and allocation of PRPs among independent sets of activated synapses. Although the nature of the synaptic tag remains unclear, it is generally accepted that it must be a local and transient molecular alteration caused by plasticity-inducing synaptic activation which can capture PRPs^8^. Several molecules have been implicated in LTP and LTD maintenance by synaptic tagging and capture mechanisms^9^. This includes calcium/calmodulin dependent kinase II (CaMKII) for LTP tagging and capture^10^,^11^,^12^ and mitogen-activated protein kinases (MAPKs) for LTD tagging and capture^12^. One confounding aspect is that upon induction of plasticity, of either LTP or LTD, the setting of the synaptic tag is intrinsically indistinguishable from the expression of synaptic plasticity. This may explain why several candidate molecules have been implicated in the setting of the synaptic tag and why particular molecules have been specifically implicated in the setting of LTP tags^13^ or LTD tags^12^,^14^. Alternatively, one can consider the synaptic tag as a remodelling of the post-synaptic density (PSD), induced by LTP or LTD rendering synapses permissive to change.

In this working model, the dynamic modulation of the cytoskeleton is a major candidate for an activity-dependent permissive state of synapses. In particular, the actin network in neurons is extremely dynamic and neuronal activity can regulate a rapid turnover of actin. Induction of synaptic plasticity can lead to the remodelling of the actin cytoskeleton at the synapse, both pre- and post-synaptically^15^,^16^,^17^, which provides a temporal and spatial activity-dependent mechanism to regulate synaptic morphology and function. Previous studies have shown that a dynamic actin cytoskeleton is required for the induction of LTP^18^,^19^ and LTD^20^. Additionally, there is evidence that the expression of persistent forms of plasticity involves the restructuring of the actin cytoskeleton, with an increase in synaptic F-actin content in LTP^16^ and a decrease in LTD^21^. Interestingly, CaMKII, a key molecule for LTP, modulates the dynamics of the actin cytoskeleton by promoting the stabilization of actin filaments and maintaining the structure of dendritic spines^22^. Upon activation, CaMKII transiently un-binds from actin, which is necessary for the re-organization of the actin filaments^23^. This suggests a transient state of actin dynamics triggered upon the induction of synaptic plasticity. In addition, CaMKII has been implicated in synaptic capture^10^,^11^,^12^ and in LTD induction^24^,^25^. Taken this into account it is plausible that activity-dependent modulation of actin through CaMKII activation underlies the setting of the LTD synaptic tag.

## Results

### Persistent forms of LTD are dependent on protein synthesis

**Figure 1:**
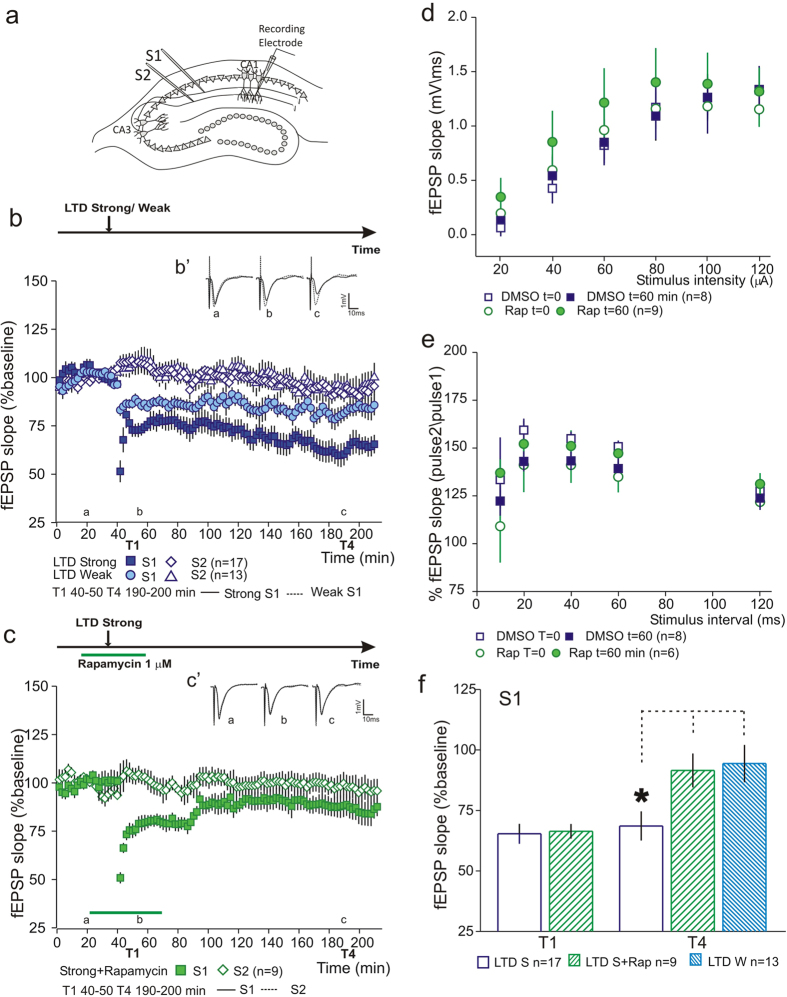
Persistent forms of LTD are dependent on protein synthesis. (**a**) Schematic representation of a hippocampal slice with the positioning of stimulating and recording electrodes. (**b**) Induction of LTD with strong low-frequency stimulation (LTD Strong – 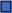) induced a persistent form of LTD whereas induction of LTD with weak low-frequency stimulation (LTD Weak – 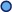) lead to a transient form which returns to baseline at the end of the recording (210 minutes). Slice viability was assessed by recording a second independent pathway that did not show a significant decrease throughout the recording (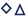). (B’) Average field excitatory postsynaptic potentials (fEPSPs) traces (average of three consecutive individual traces) for LTD strong (black) and LTD Weak (dashed) conditions at times indicated; scale −1 mV, 10 ms. (**c**) Inhibition of protein synthesis by Rapamycin application (1 μM – 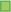) at the time of LTD induction, blocked the maintenance of LTD. No effect of Rapamycin was observed in slice viability (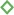 control pathway – S2). (C’) Average field excitatory postsynaptic potentials (fEPSPs) traces (average of three consecutive individual traces) for Rapamycin S1 (black) and S2 (dashed) at times indicated; scale −1 mV, 10 ms. (**d**) Summary plot showing the percentage of fEPSP slope at T1 (40–50 min) and T4 (190–200 min), normalized by the percentage change of the control pathway. The application of Rapamycin blocked the induction of LTD. n = number of slices. (**e**) Rapamycin or DMSO application did not alter PPF. (**f**) I/O curve for DMSO and Rapamycin. Drugs were bath-applied for 1 h similarly to the electrophysiology experiments. No significant difference was obtained for all tested conditions.

Previous studies have suggested that persistent forms of plasticity such as long-term depression (LTD) required the synthesis of plasticity-related proteins^26^. As mentioned above, the synaptic tagging and capture (STC) allows a transient form of plasticity (early-LTD or E-LTD) to be maintained through the capture of PRPs that become available by the induction of a persistent form of plasticity (late-LTD or L-LTD) in an independent set of synapses^8^. We set out to determine whether transient and persistent forms of LTD were induced by different stimulation paradigms (see methods and ref. 26). Using a strong low-frequency stimulation (strong LFS) we were able to induce an L-LTD that was stable until the end of the recording (LTD-Strong/S1 – Fig. 1b). Conversely, synaptic stimulation with a weak low-frequency stimulation (Weak LFS) results in an E-LTD that returns to baseline values at the end of the recording (LTD-Weak/S1 - Fig. 1b). To test whether L-LTD was dependent on *de novo* protein synthesis, Rapamycin (1 μM), an mTOR-dependent protein synthesis inhibitor^27^, was bath-applied at the time of strong LTD induction. We observed that inhibition of protein synthesis blocks the induction of L-LTD (Strong + Rapamycin/S1 Fig. 1c). In all experiments, a second independent pathway (S2) was monitored throughout the experiment, and no significant change in synaptic transmission was observed in all tested conditions (Fig. 1b,c, open symbols). Statistical analysis of LTD in all tested conditions was performed by comparing LTD values at T1 (40–50 min) and T4 (190–200 minutes), normalized by the percentage change from baseline values in the control pathway. We observed that inhibition of protein synthesis did not interfere with the induction of LTD but significantly reduced the expression of LTD at the end of the recording (T1 LTD S 66.4 ± 3.04% n = 17; LTD S + Rap 65.35 ± 4.11% n = 9; F(1, 24) = 0.03 P = 0.86; T4 LTD S 68.6 ± 6.03% n = 17; LTD W 94.4 ± 7.72% n = 13; LTD S + Rap 91.54 ± 7.02% n = 9; F(2, 36) = 4.64 P = 0.02; Fisher *post hoc* test, LTD W vs LTD S *P = 0.008; LTD S + Rap vs LTD S *P = 0.03; LTD W vs LTD S + Rap P = 0.79; Fig. 1d). To rule out the unspecific effect of Rapamycin or DMSO application on the electrophysiological properties of hippocampal neurons we analyzed input-output (I/O) curves and pair-pulse facilitation (PPF) in Rapamycin and DMSO-treated slices. No significant change was observed before and after drug application (1 hour) or between tested conditions (Fig. 1e,f). These results indicate that different stimulation paradigms can induce transient (E-LTP) and persistent forms of LTD (L-LTD) and that persistent forms of LTD are protein synthesis dependent.

### The induction of a persistent form of LTD is dependent on a dynamic actin cytoskeleton

**Figure 2:**
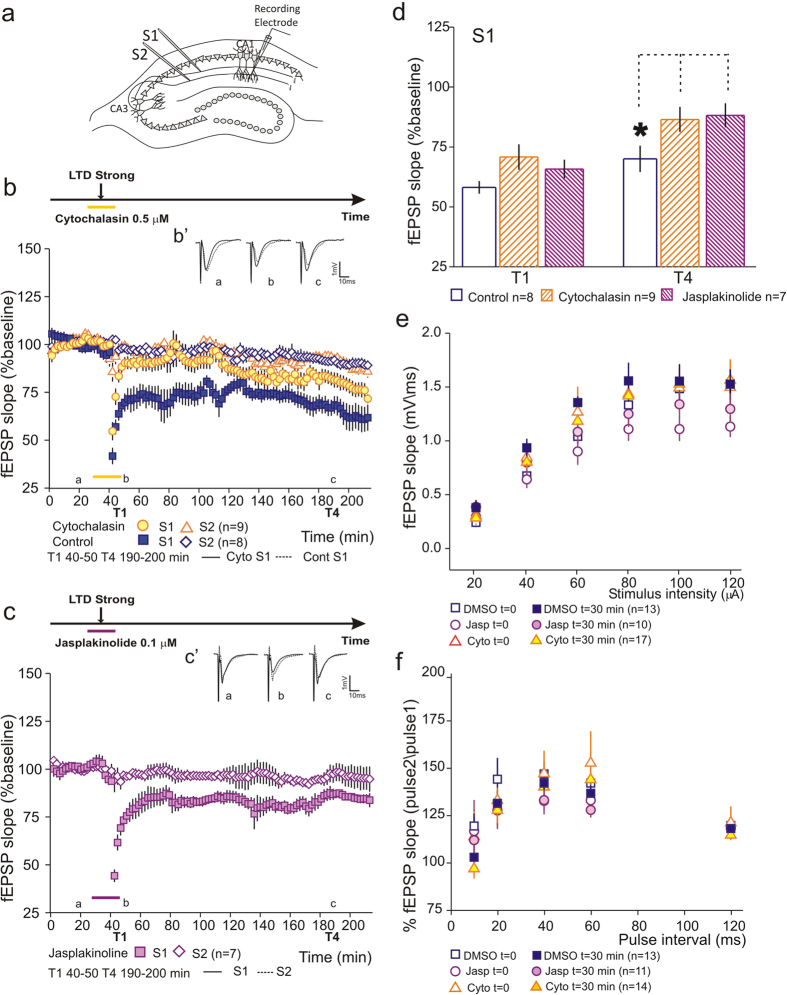
Inhibition of actin polymerization or actin depolymerization blocks LTD induction. (**a**) Schematic representation of a hippocampal slice with the positioning of stimulating and recording electrodes. (**b**) Application of Cytochalasin-B (0.5 μM – 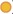) for 30 minutes during LTD induction blocked LTD induction as compared to control conditions (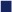). Drug application started 10 minutes prior to LTD induction and was washed out 5 minutes post-induction. No effect of Cytochalasin was observed in the control pathway throughout the recorded time (S2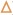). (B’) Average field excitatory postsynaptic potentials (fEPSPs) traces (average of three consecutive individual traces) for Cytochalasin (black) and Control (dashed) conditions at times indicated; scale −1 mV, 10 ms. (**c**) Application of Jasplakinolide (0.1 μM – 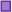) also blocked LTD induction. Drug application was similar to Cytochalasin application described above. No effect of Jasplakinolide was observed in the control pathway throughout the recorded time (S2 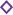). (C’) Average field excitatory postsynaptic potentials (fEPSPs) traces (average of three consecutive individual traces) for Jasplakinolide S1 (black) and S2 (dashed) at times indicated; scale −1 mV, 10 ms (**d**) Summary plot showing the percentage fEPSP slope at the time window T1 and T4. The application of Cytochalasin or Jasplakinolide blocked LTD induction as compared to controls; n = number of slices. (**e**) DMSO, Cytochalasin or Jasplakinolide application does not alter PPF. (**f**) I/O curve for DMSO, Cytochalasin and Jasplakinolide. Drugs were bath-applied for 30 minutes similarly to the LTD experiments. No significant difference was obtained for all tested conditions.

We have previously shown that interfering with dynamics of the actin cytoskeleton blocks the induction of persistent forms of LTP^11^. To test the role of the actin cytoskeleton dynamics in LTD we bath-applied either an actin polymerization inhibitor, Cytochalasin (0.5 μM), or an actin depolymerization inhibitor, Jasplakinolide (0.1 μM), at the time of LTD induction. Cytochalasin application for 30 minutes during LTD induction blocked the induction of L-LTD (S1 - Fig. 2b), as compared to control slices. Similarly, Jasplakinolide application for 30 minutes during LTD induction also leads to a blockade of L-LTD (S1 - Fig. 2c). Drug application had no impact in synaptic transmission nor in slice viability as assessed by recording a second independent pathway (S2 - Fig. 2b,c, open symbols). Statistical analysis of LTD at T1 (40–50 min) and T4 (190–200 minutes), normalized by the control pathway, shows that Cytochalasin or Jasplakinolide application did not interfere with LTD induction (T1 Control 58.3 ± 2.63% n = 8; Cytochalasin 70.97 ± 5.31 n = 9; Jasplakinolide 64.94 ± 3.9 n = 7; F(2, 21) = 2.35 P = 0.12), but significantly decreased LTD values at the end of the recording (T4 Control 70.18 ± 5.51% n = 8; Cytochalasin 86.62 ± 5.25 n = 9; Jasplakinolide 88.36 ± 5.01 n = 7; F(2, 21) = 3.52 P = 0.048; Fisher *post hoc* test, Cytochalasin vs control *P = 0.035; Jasplakinolide vs control *P = 0.029; Cytochalasin vs Jasplakinolide P = 0.82 Fig. 2d). We did not find any effect of Cytochalasin or Jasplakinolide application on the electrophysiological properties of hippocampal neurons analyzed by input-output (I/O) curves and pair-pulse facilitation (PPF). No significant change was observed before and after drug application (30 min) or between tested conditions (Fig. 2e,f). These results show that, similarly to LTP, the induction of a persistent form of LTD is dependent on a dynamic actin cytoskeleton.

### The maintenance of LTD is blocked by inhibition of actin depolymerization

**Figure 3:**
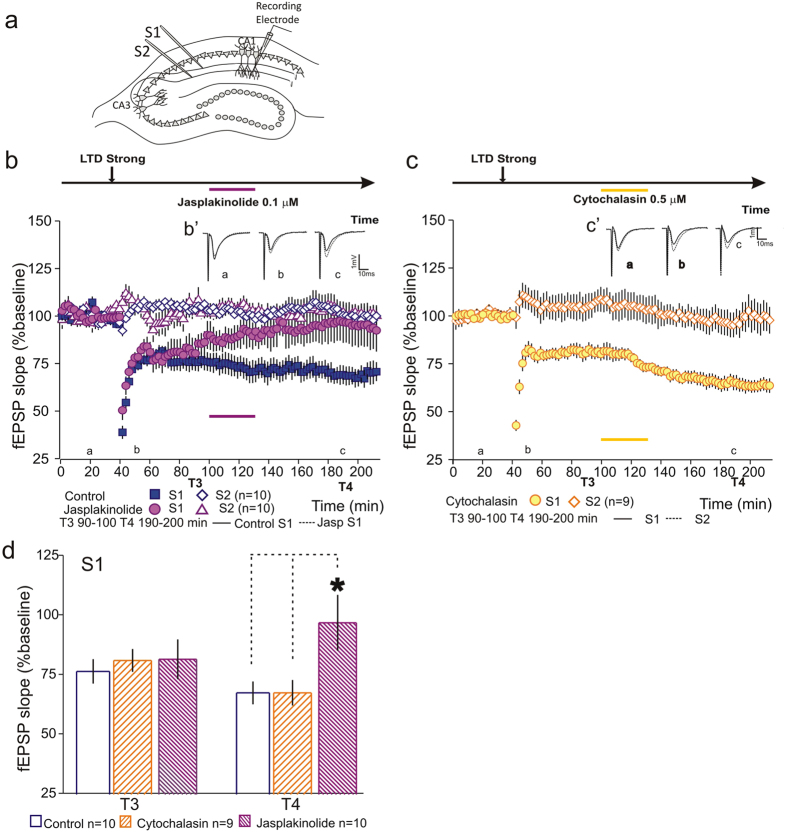
Inhibition of actin depolymerization blocks the maintenance of LTD. (**a**) Schematic representation of a hippocampal slice with stimulating and recording electrodes. (**b**) Application of Jasplakinolide (0.1 μM – 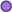), for 30 minutes, one hour after LTD induction, blocked LTD maintenance, as compared to controls (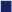). No effect of Jasplakinolide was observed in slice viability (S2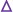). (B’) Average field excitatory postsynaptic potentials (fEPSPs) traces (average of three consecutive individual traces) for Control (black) and Jasplakinolide (dashed) at times indicated; scale −1 mV, 10 ms. (**c**) Application of Cytochalasin-B (0.5 μM – 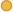) for 30 minutes during LTD maintenance did not interfere with LTD. No effect of Cytochalasin was observed in slice viability (S2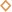). (C’) Average field excitatory postsynaptic potentials (fEPSPs) traces (average of three consecutive individual traces) for Cytochalasin (black) and Control (dashed) conditions at times indicated; scale −1 mV, 10 ms. (**d**) Summary plot showing the percentage fEPSP slope at T3 and T4. The application of Jasplakinolide blocked LTD maintenance as compared to Control and Cytochalasin-treated slices; n = number of slices.

Previous reports have shown that the induction of a persistent form of LTP induces a net increase in filamentous actin (F-actin), whereas the induction of LTD induces a net decrease in F-actin^28^. To test whether inhibition of actin dynamics blocks the maintenance of LTD we applied either an actin polymerization inhibitor, Cytochalasin (0.5 μM) or an actin depolymerization inhibitor, Jasplakinolide (0.1 μM), for 30 minutes during LTD maintenance. While Cytochalasin application did not interfere with LTD maintenance (S1- Fig. 3c), Jasplakinolide application blocked LTD maintenance (S1- Fig. 3b). As before, drug application had no impact in synaptic transmission or slice viability (S2 - Fig. 3b,c, open symbols). Statistical analysis of LTD at T3 (100–110 min) and T4 (190–200 minutes), normalized by the control pathway, shows that LTD expression was similar in all tested conditions before drug application (T3 Control 74.4 ± 5.16% n = 10; Cytochalasin 81.3 ± 4.78 n = 9; Jasplakinolide 81.8 ± 8.37 n = 10; F(2, 26) = 0.19 P = 0.83), but was significantly decreased if actin depolymerization was blocked (T4 Control 67.7 ± 4.8% n = 10; Cytochalasin 67.8 ± 5.36 n = 9; Jasplakinolide 97.15 ± 11.7 n = 10; F(2, 26) = 4.46 P = 0.02; Tukey *post hoc* test, Jasplakinolide vs Control *P = 0.038; Jasplakinolide vs Cytochalasin *P = 0.044; Cytochalasin vs Control P = 0.99 Fig. 3d). These results show that contrary to what we observed for LTP, the maintenance requires the depolymerization of actin, suggesting that LTD is associated with a net decrease in F-actin content.

### Inhibition of actin depolymerization blocks synaptic capture

**Figure 4:**
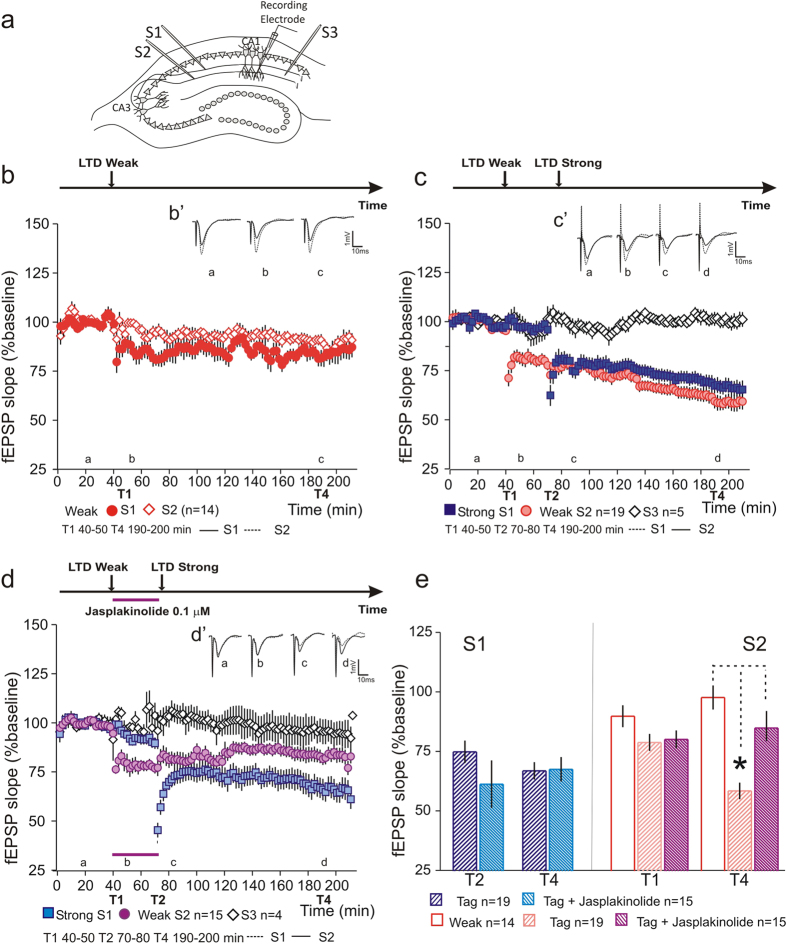
Inhibition of actin depolymerization blocks synaptic capture. (**a**) Schematic representation of a hippocampal slice with the positioning of stimulating (S1, S2 and S3) and recording electrodes. (**b**) Weak LFS lead to a transient form of LTD (Weak – 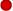). (B’) Average field excitatory postsynaptic potentials (fEPSPs) traces (average of three consecutive individual traces) for Weak S1 (black) and S2 (dashed) at times indicated; scale −1 mV, 10 ms. (**c**) Induction of a persistent form of LTD in a second independent pathway (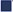) is able to maintain the transient E-LTD (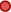) – synaptic capture. Slice viability was assessed by recording a third pathway that did not show a significant decrease throughout the recording (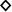). (C’) Average field excitatory postsynaptic potentials (fEPSPs) traces (average of three consecutive individual traces) for weak (black) and strong (dashed) conditions at times indicated; scale −1 mV, 10 ms. (**d**) Application of Jasplakinolide (0.1 μM) for 30 minutes between Weak LTD (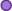) and Strong LTD (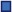) stimulation blocked synaptic-tagging and capture. No effect of Jasplakinolide was observed in slice viability (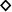). (D’) Average field excitatory postsynaptic potentials (fEPSPs) traces (average of three consecutive individual traces) for Weak S2(black) and Strong S1(dashed) conditions at times indicated; scale −1 mV, 10 ms. (**e**) Summary plot showing the percentage fEPSP slope after LTD induction, T1 and T2, and at the end of the recording T4. The application of Jasplakinolide significantly increased LTD values in S2 as compared to control synaptic-tagging and capture condition. No difference was obtained in the LTD expression induced by strong LFS between Jasplakinolide and controls; n = number of slices.

If the modulation of actin dynamics underlies the synaptic tag, then interfering with the dynamics of actin cytoskeleton should impair the capture of PRPs irrespectively of whether LTP or LTD is induced. To test this hypothesis, we performed an LTD synaptic tagging and capture experiment while simultaneously inhibiting actin depolymerization. As previously shown, synaptic stimulation with a weak LFS leads to the induction of an E-LTD which returns to baseline values at the end of the recording (Fig. 4b). Conversely, if 30 minutes after the weak LFS, an L-LTD is induced by strong LFS stimulation of a second independent set of synapses, the E-LTD is maintained by synaptic tagging and capture associativity (Fig. 4c). If actin depolymerization is inhibited, by Jasplakinolide application during the time interval between weak LFS and strong LFS (30 minutes), synaptic capture is blocked (Fig. 4d). Statistical analysis of LTD after stimulation (T1 and T2: 70–80 min) and at the end of recording (T4), normalized by the control pathway, shows that although initial E-LTD was similar in all tested conditions (T1 S2-Weak 89.95 ± 4.58% n = 14; Tag 78.96 ± 3.55% n = 19; Tag + Jasp 80.26 ± 3.76% n = 15; F(2, 45) = 2.2, P = 0.12), LTD values were significantly lower in the synaptic tagging and capture condition (T4 S2-Weak 97.82 ± 5.05% n = 14; Tag 58.63 ± 3.48% n = 19; Tag + Jasplakinolide 85.01 ± 13.48% n = 15; F(2, 45) = 26.52, P = 0.0001; Tukey *post hoc* test, Weak vs Tag *P = 0.0001; Tag vs Tag + Jasplakinolide *P = 0.0001; Weak vs Tag + Jasplakinolide P = 0.09 Fig. 4e). No difference was observed in LTD induced by strong LFS between Jasplakinolide and control conditions (T2 S1-Tag 75.03 ± 4.7% n = 19; Tag + Jasplakinolide 61.44 ± 9.9% n = 15; F(1, 32) = 4.78, P = 0.053; T4 S1- Tag 67.05 ± 3.65% n = 19; Tag + Jasplakinolide 67.69 ± 5.12% n = 15; F(1, 32) = 0.01, P = 0.92; Fig. 4e). This is due to the fact that drug application is suspended before strong LFS, which lasts 15 minutes, combined with a fast slice perfusion system. Our results show that, similarly to what we found for LTP, a dynamic actin cytoskeleton is necessary for LTD synaptic tagging and capture.

### CaMKII activation is necessary for LTD synaptic tagging and capture

**Figure 5:**
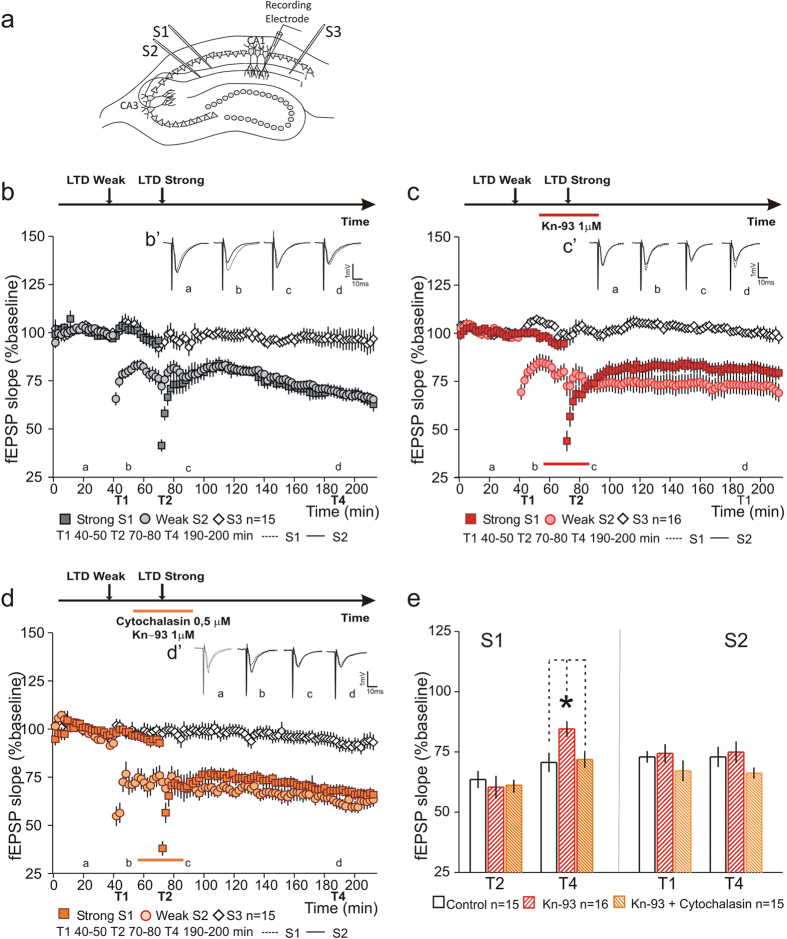
Inhibition of actin polymerization rescues the impairment in synaptic capture induced by CaMKII inhibition. (**a**) Schematic representation of a hippocampal slice with stimulating and recording electrodes. (**b**) Induction of a persistent LTD, by strong LFS (S1 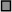), leads to the maintenance of a transient form of LTD, induced by a weak LFS (S2 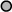). No changes were observed in S3 (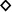). (B’) Average fEPSP for S2 (black) and S1 (dashed) at times indicated; scale −1 mV, 10 ms. (**c**) Inhibition of CaMKII by Kn-93 (1 μM) application did not blocked the synaptic capture of PRPs in S2 (Weak 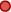), but reduced the expression of LTD in the strong stimulated S1 (Strong 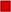). Kn-93 application had no impact in slice viability (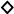). (C’) Average fEPSPs for S2 (black) and S1 (dashed) at times indicated; scale −1 mV, 10 ms. (**d**) Inhibition of actin polymerization by Cytochalasin application, co-applied with Kn-93 (1 μM), restored the expression of LTD (Strong S1 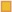) to control levels, while having no impact in the maintenance of the transient LTD (Weak S2 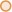). Cytochalasin and Kn-93 co-application had no impact in slice viability (S3 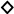). (D’) Average fEPSPs traces for S2 (black) and S1 (dashed) at times indicated; scale −1 mV, 10 ms. (**e**) Summary plot showing the percentage fEPSP slope at T1, T2 and T4. The inhibition of CaMKII by Kn-93 application reduced LTD expression in S1. Co-application with Cytochalasin restored LTD to control values. LTD values in S2 were not significantly different between tested conditions. n = number of slices.

Previous studies have indicated a critical role of CaMKII activation in the setting of an LTP synaptic tag^10^,^12^. Additionally, we found that inhibiting actin polymerization is sufficient to rescue the impairment in LTP synaptic capture induced by CaMKII inhibition^11^. Since CaMKII is also implicated in LTD induction^24^,^25^ and its activity has a direct impact in the modulation of the actin cytoskeleton, we set out to test whether CaMKII activation is also required for LTD synaptic tagging and capture. As in previous studies, we use Kn-93, an inhibitor of CaMKII activation that, in a dose-dependent manner, inhibits CaMKII activity leaving intact the phosphorylation of CREB and downstream induction of gene expression and the PRPs synthesis^10^. We found that CaMKII inhibition, by Kn-93 (1 μM) application, reduced the expression of L-LTD (S1), compared to controls, while allowing the maintenance of the E-LTD (S2) by synaptic-tagging and capture (Fig. 5a,c). Kn-93 was bath-applied for a total of 40 minutes, starting 15 minutes prior to induction of L-LTD (strong LTD). Interestingly, and similar to what we found in LTP synaptic-tagging and capture, inhibition of actin polymerization rescued the effect of CaMKII inhibition in LTD tagging and capture. Co-application of Cytochalasin (0.5 μM) with Kn-93 (1 μM) restored the expression of the L-LTD (S1) to control values (Fig. 5b,d). Statistical analysis of LTD after stimulation (T1 and T2) and at the end of recording (T4), normalized by the control pathway, shows that L-LTD expression is significantly blocked in Kn-93 treated slices as compared to Kn-93 + Cytochalasin treated slices or controls (T2 S1-Control 63.63 ± 3.57% n = 15; Kn-93 60.47 ± 4.66% n = 16; Kn-93 + Cytochalasin 61.3 ± 2.57% n = 15; F(2, 43) = 0.14, P = 0.87; T4 S1-Control 70.68 ± 3.97% n = 15; Kn-93 84.55 ± 3.24% n = 16; Kn-93 + Cytochalasin 71.95 ± 3.53% n = 15; F(2, 43) = 4.68, P = 0.01; Tukey *post hoc* test, Control vs Kn-93 *P = 0.023; Kn-93 vs Kn-93 + Cytochalasin *P = 0.04; Control vs Kn-93 + Cytochalasin P = 0.97 Fig. 5e). No difference was observed in LTD for S2 in all conditions tested (T1 S2-Control 72.9 ± 2.4%, n = 15; Kn-93 74.45 ± 3.86%, n = 16; Kn-93 + Cytochalasin 67.22 ± 4.28%, n = 16, F(2, 43) = 1.16, P = 0.32; T4 S2-Control 72.98 ± 4.23%, n = 15; Kn-93 74.99 ± 4.41%, n = 16; Kn-93 + Cytochalasin 66.26 ± 2.32%, n = 16; F(2, 43) = 1.44, P = 0.25; Fig. 5e). Our results show that CaMKII activation has a role in LTD synaptic tagging and capture. Moreover, since the inhibition of actin polymerization rescued the impairment in LTD expression induced by CaMKII inhibition, our results suggest that CaMKII activation is involved in the modulation of actin dynamics.

### Activity-dependent modulation of actin dynamics mimics the setting of an LTD synaptic tag

**Figure 6:**
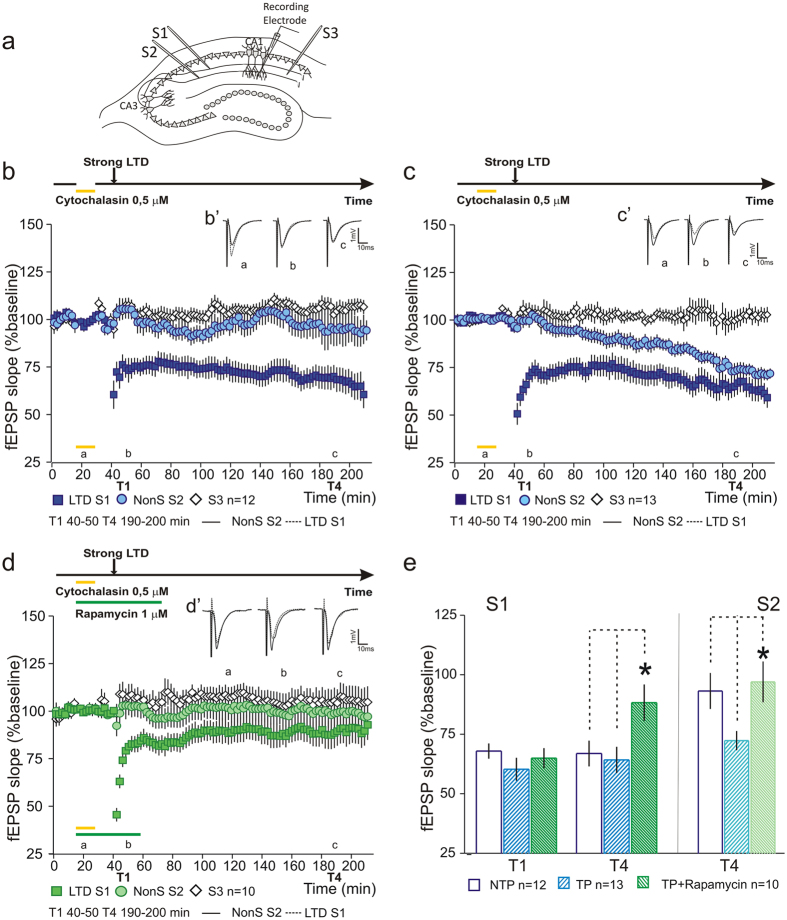
Modulation of actin dynamics mimics the setting of a LTD synaptic tag. (**a**) Schematic representation of a hippocampal slice with stimulating and recording electrodes. (**b**) Cytochalasin application (15 minutes during baseline) had no effect on LTD induction and maintenance (LTD 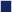) or on the NonS pathway (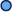). No effect of Cytochalasin was observed in slice viability (S3 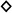). (B’) Average fEPSPs for NonS (S2-black) and LTD (S1-dashed) at times indicated; scale −1 mV, 10 ms. (**c**) Synaptic activation in the NonS pathway (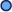) during the Cytochalasin application was sufficient to induce the expression of LTD. Cytochalasin application has no effect on LTD induction and maintenance (LTD 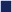) nor in slice viability (S3 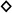). (C’) Average fEPSPs for NonS (S2-black) and LTD (S1-dashed) at times indicated; scale −1 mV, 10 ms. (**d**) Inhibition of protein synthesis by Rapamycin application blocked LTD induction (LTD 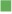) as well as the expression of LTD in S2 (NonS 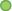). No effect of Cytochalasin and Rapamycin was observed in S3 (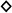). (D’) Average fEPSPs for NonS (S2- black) and LTD (S1- dashed) conditions at times indicated; scale −1 mV, 10 ms. (**e**) Summary plot showing the percentage fEPSP slope at T1 and T4. Inhibition of actin polymerization leads to the expression of LTD in a non-stimulated pathway, which is dependent on protein synthesis. LTD induced in the presence of Rapamycin is significantly reduced. n = number of slices.

The experiments described above support our hypothesis that modulation of actin dynamics is involved in the setting of the synaptic tag, presumably through CaMKII activation. It is, thus plausible that pharmacological modulation of actin dynamics is sufficient to set a synaptic tag that allows non-stimulated synapses to express LTD by tagging and capture associativity. To test this, we designed an experiment in which polymerization of actin is inhibited during baseline. In this design, one of the pathways (S1) was stimulated with a strong LFS (LTD – S1). A second pathway was used as a test pathway and did not receive any plasticity-inducing stimuli (Non-stimulated pathway - NonS/S2). Additionally, we also recorded a third, retrograde stimulated pathway that was used as a control pathway (S3). Since we previously observed that the effect of actin modulation in LTP is dependent on neuronal activation^11^, two designs were considered. In the first set of experiments, the non-test pulse design (NTP), the test pulse stimulation in S2, was suspended during Cytochalasin application (0.5 μM; 15 minutes). Also, test pulse stimulation was suspended in the control pathway (S3), during drug application. We found that Cytochalasin application did not interfere with the induction of L-LTD (S1) nor did it induced any significant change from baseline levels in S2 or S3 (Fig. 6b). In the second set of experiments, test pulse design (TP), the test pulse stimulation was present in S2 during Cytochalasin application. We found that Cytochalasin application, in an activity-dependent manner, lead to the expression of LTD in non-stimulated synapses (S2 - Fig. 6c). This expression of LTD in non-stimulated synapses was due to the capture of PRPs synthesized upon L-LTP induction in S1. If protein synthesis was inhibited, by concomitant Cytochalasin and Rapamycin application, the expression of L-LTD in S1 was blocked as well as the LTD in S2 (Fig. 6d). Rapamycin was applied for one hour and co-applied with Cytochalasin for 15 minutes during baseline recording (timeline on top of Fig. 6d). Statistical analysis of LTD at times indicated (T1 and T4), normalized by the control pathway, shows that LTD in S1 is significantly lower in Rapamycin treated slices (T1 S1-NTP 68.11 ± 3.21% n = 12; TP 60.47 ± 4.84% n = 13; TP + Rapamycin 65.14 ± 4.23% n = 10; F(2, 32) = 1.2, P = 0.32; T4 S1-NTP 67.09 ± 5.42% n = 12; TP 64.4 ± 5.49% n = 13; TP + Rapamycin 88.43 ± 7.64%, n = 10; F(2, 32) = 4.32, P = 0.02; Fisher *post hoc* test, NTP vs TP + Rapamycin *P = 0.023; TP vs TP + Rapamycin *P = 0.009; NTP vs TP P = 0.74 Fig. 6e). LTD expression in the non-stimulated pathway (S2) was significantly lower in the condition when test-pulse stimulation was paired with Cytochalasin application (T4 S2-NTP 93.35 ± 7.55%, n = 12; TP 72.49 ± 4.05%, n = 13; TP + Rapamycin 97.17 ± 8.6%, n = 10, F(2, 32) = 3.91, P = 0.03; Fisher *post hoc* test, Control NTP vs Control TP *P = 0.03; Control TP vs TP Rapamycin *P = 0.01; Control NTP vs TP Rapamycin P = 0.7 Fig. 6e). Our results show that blocking actin polymerization, in an activity-dependent manner, can set a synaptic tag that captures PRPs and allows the expression of LTD in non-stimulated synapses.

### Time-course of tag activity is dependent on neuronal activity

**Figure 7:**
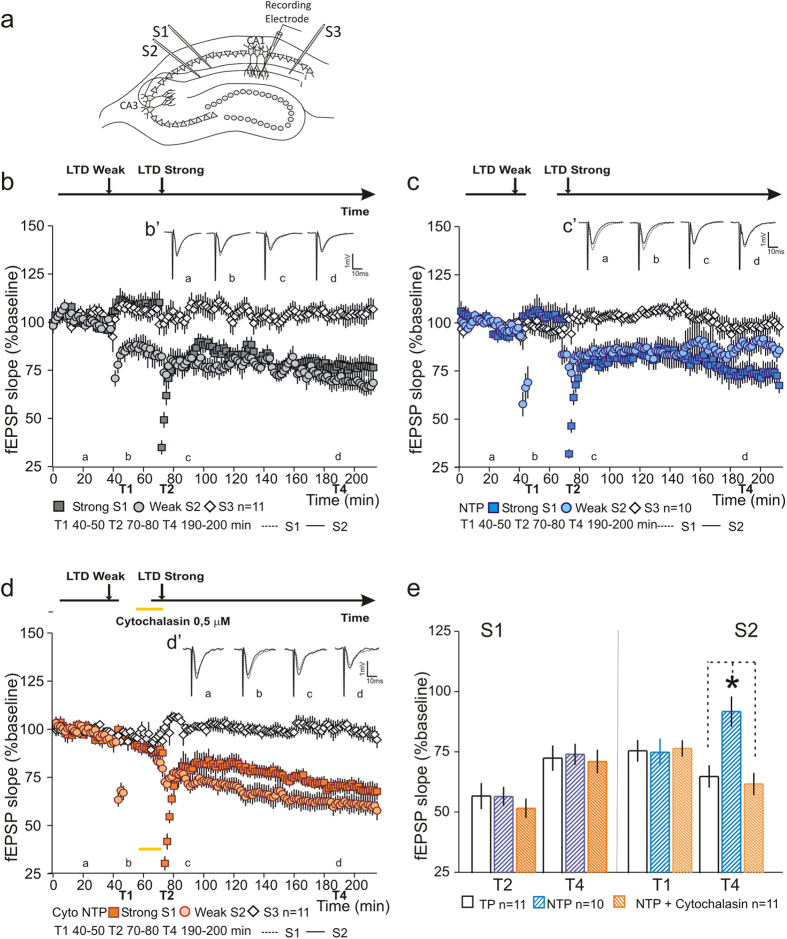
Duration of the synaptic tag is activity dependent. (**a**) Schematic representation of a hippocampal slice with stimulating and recording electrodes. (**b**) A transient form of LTD (Weak S2 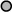) is maintained by synaptic-tagging and capture (Strong S1 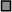). Synaptic transmission was stable throughout the recording (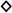). (B’) Average fEPSPs for S2 (black) and S1 (dashed) at times indicated; scale −1 mV, 10 ms. (**c**) Suspending test-pulse stimulation after weak LFS had no impact in the expression of the transient form of LTD but blocked the synaptic capture of PRPs (NTP S2 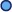). No difference was observed in the expression of strong LTD (S1 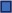) nor in slice viability (S3 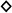). (C’) Average fEPSPs for S2 (black) and S1 (dashed) at times indicated; scale −1 mV, 10 ms. (**d**) Inhibition of actin polymerization restores synaptic capture. Cytochalalsin (0.5 μM) was applied for 15 minutes immediately before strong LFS (Cyto NTP S1 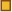) and paired with 5 minutes of test-pulse stimulation in the weak LFS pathway (Cyto NTP S2 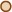). Synaptic transmission was stable throughout the recording (S3 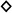). (D’) Average fEPSPs for S2 (black) and S1 (dashed) at times indicated; scale −1 mV, 10 ms. (**e**) Summary plot showing the percentage fEPSP slope at T1, T2 and T4. Suspending test-pulse stimulation significantly decreased the expression of LTD in the Weak LFS pathway (NTP S2). Expression of LTD was restored to Control levels (TP S2) by application of an actin polymerization inhibitor (NTP + Cytochalasin S2). LTD expression in the strong LFS pathway (S1) was not significantly different between tested conditions. n = number of slices.

One of the critical aspects of the synaptic tag is its duration. If the synaptic tagging and capture model provides a cellular and molecular mechanism for synaptic integration, then the duration of the tag will determine the time-course of synaptic integration. Previous studies have reported a time course for the LTP tag of about 1 hour^29^. However, previous experiments using electrophysiological recordings, have recorded synaptic responses during the whole duration of the experiment at a constant frequency. Although the test-pulse stimulation is necessary to access fEPSP slope it is clear that, even at very low frequencies, test-pulse stimulation alters the turn-over of PRPs and synaptic dynamics^30^,^31^. To test whether test-pulse stimulation alters the time-course of the synaptic tag, we performed an LTD synaptic tagging and capture experiment in which the test pulse was suspended during the time-interval between weak and strong LFS (NTP). In this case, E-LTD was not maintained by synaptic-tagging and capture associativity (S2 Fig. 7b,c). Test-pulse stimulation applied to S2 was restricted to 5 minutes after weak LFS and to 5 minutes immediately before strong LFS. Suspending test pulse stimulation had no impact on the initial expression of LTD or in LTD expression at the time point immediately before strong LFS (S2 - Fig. 7b,c). Cytochalasin (0.5 μM) application did not interfere with the induction of L-LTD in S1 and was sufficient to restore the maintenance of E-LTD by synaptic-tagging and capture. Cytochalasin was applied for 15 minutes before strong LFS stimulation of S1 and was paired with test-pulse stimulation of S2 during 5 minutes before strong LFS stimulation (Fig. 7d – see insert for time frame). Statistical analysis of LTD at times indicated (T1, T2 and T4), normalized by the control pathway, shows that LTD in S2 is significantly higher when test-pulse stimulation is suspended as compared to Cytochalasin treated slices or control conditions (T1 S2-TP 75.73 ± 4.49% n = 11; NTP 75.15 ± 5.73% n = 10; NTP + Cytochalasin 76.77 ± 3.37% n = 11; F(2, 29) = 0.37, P = 0.71; T4 S2-TP 65.1 ± 4.64% n = 11; NTP 92.04 ± 6.27% n = 10; NTP + Cytochalasin 62.01 ± 4.6% n = 11; F(2, 29) = 10.5, P = 0.00037; Tukey *post hoc* test, TP vs NTP *P = 0.002; NTP vs NTP + Cytochalasin *P = 0.0007; TP vs NTP + Cytochalasin P = 0.89 Fig. 7e). LTD expression in S1 was similar for all tested conditions (T2 S1-TP 56.98 ± 5.4% n = 11; NTP 56.81 ± 4.03%, n = 10; NTP + Cytochalasin 51.9 ± 4.03% n = 11; F(2, 29) = 0.46, P = 0.64; T4 S1-TP 72.0 ± 5.26% n = 11; NTP 74.29 ± 4.39% n = 10; NTP + Cytochalasin 71.35 ± 4.89%, n = 11; F(2, 29) = 0.09, P = 0.91; Fig. 7e). Our results show that the ability to capture PRPs, e.g. the duration of the synaptic tag, is activity-dependent through the modulation of the synaptic actin cytoskeleton.

## Discussion

The synaptic tagging and capture model provides a conceptual framework that allows conciliating how synaptic plasticity maintains its input specificity while depending on macromolecules that are distributed cell-wide. It proposes that the maintenance of long-lasting forms of plasticity depends on the interplay between synapse-specific tags and plasticity-related proteins (PRPs). This allows the association of separated synaptic events, within large time windows, through the cooperative allocation of PRPs among activated synapses. This form of synaptic cooperation depends on the duration of the synaptic tag and also on the availability of PRPs^8^. Since the initial description of the synaptic tag by Frey and Morris^6^, several studies have been conducted to understand the nature of the synaptic tag and the rules underlying this mechanism of synaptic associativity. While several molecules have been implicated in LTP and LTD synaptic tagging, we favor a model in which the synaptic tag is the state of the synapse, which can be permissive or not to modification. In this context, an input-specific modulation of synaptic actin may represent these two synaptic states, permissive or not permissive. The role of actin cytoskeleton in regulating synaptic plasticity has been well described^32^,^33^. In addition, there are clear indications that a dynamic actin cytoskeleton is involved in the setting of the LTP synaptic tag^11^. Here, we show that LTD maintenance is dependent on *de novo* protein synthesis, similarly to what has been described for LTP^11^. We also show that the induction of L-LTD requires a dynamic actin cytoskeleton. Consistent with previous studies^34^, we found that the expression of LTD is blocked by inhibition of either actin polymerization or depolymerization. Interestingly, we found that LTD maintenance is blocked by inhibition of actin depolymerisation but not by inhibition of actin polymerization, pointing out to a different modulation of actin dynamics upon induction of plasticity versus its maintenance. The observation that LTD induction is blocked by inhibition of actin depolymerization is consistent with the observation that LTD induction is associated with AMPA receptor internalization^35^ and actin re-organization through cofilin modulation^28^. Moreover, the blockade of LTD maintenance by inhibition of actin depolymerization goes in line with the hypothesis, already described in previous studies, that the expression of persistent forms of LTD is associated with a decrease in F-actin content in spines^21^. Taking this into account, the observation that LTD induction is also blocked by inhibition of actin polymerization is, perhaps, counter-intuitive. One possible explanation for this observation relies on the existence of different populations of actin in spines, with different structural forms, dynamics and life-times^36^,^37^. Actin filaments present at spine heads are mesh-like structures with a rapid turnover versus the thick filaments extending from the dendritic shaft into the spine neck. It is possible that the effect we observed in LTD induction is due to a blockade in the dynamics of the high-dynamic actin filaments and reflects an impairment in a rapid and transient re-organization of synaptic actin. Conversely, the blockade in LTD maintenance, induced by inhibiting actin depolymerization, is due to an artificial increase in the low-dynamic actin filaments. It remains to be tested how plasticity alters the dynamics of these two different synaptic actin filament populations^21^. Our results support the hypothesis that the induction of synaptic plasticity triggers an increase in actin dynamics, acting as a gating mechanism, irrespectively of whether LTD or LTP is induced. Recently, this function of gating of the actin cytoskeleton in LTP induction and maintenance was described^23^. The authors found that induction of synaptic plasticity lead to the unbinding of CaMKII from the actin cytoskeleton, opening a time window for synapse remodeling. Interestingly, this initial unbinding is permissive for the induction of LTP, when paired with synaptic activation, but rather short. The authors showed that 1 minute after LTP induction, CaMKII re-binds and stabilizes the newly formed filaments of actin, a step critically necessary for the maintenance of LTP^23^.

Since actin dynamics can be modulated locally by synaptic activation, we next tested whether temporally restricted activity-dependent modulation of actin dynamics can provide a molecular synaptic signature allowing an input specific localization of PRPs. Using a synaptic tagging and capture experimental design, we show that inhibition of actin depolymerisation blocks the maintenance of a transient LTD by synaptic tagging and capture associativity. A similar result was described previously for LTP synaptic-tagging and capture^11^, supporting our hypothesis that modulation of actin dynamics is required for the synaptic capture of PRPs. Interestingly, we found that CaMKII activation is involved, at least partially, in LTD synaptic-tagging and capture through the modulation of actin. By using a low concentration of a CaMKII inhibitor we were able to dissociate its role in synaptic tagging from the induction of PRPs synthesis^10^,^11^. CaMKII inhibition resulted in a significant reduction in LTD, compared to controls, but it did not block LTD expression completely. This may be due to parallel signalling pathways being recruited upon LTD induction that are independent from CaMKII activation^12^. Although the role of CaMKII in LTD induction and maintenance is documented^38^, our results contrast with previous observations in which the inhibition of CaMKII activation had no impact in LTD synaptic capture^12^. One possible explanation is that the age of the animals used in our study results in the induction of a form of LTD that is more dependent on CaMKII activation^39^. Despite the moderate contribution of CaMKII in LTD synaptic-tagging and capture, the observation that concomitant actin polymerization inhibition restores LTD expression to control values, suggests that the modulation of actin is triggered by CaMKII activation. Again, this supports the role of the actin remodelling as the synaptic tag. Additionally, we were able to induce a synaptic tag by inhibition of actin polymerization paired with synaptic activation. This pharmacologically induced tag was able to capture PRPs, synthesised by the induction of L-LTP in an independent set of synapses. Since synaptic activation was required for the setting of this artificial synaptic tag, it likely has a role beyond the modulation of actin filaments. Previously, we have found that NMDA receptor activation is necessary for the blockade of LTP maintenance by inhibition of actin polymerization^11^. Since NMDA receptor activation increases the turnover of synaptic proteins^30^,^40^, it is plausible that synaptic activity is necessary to accelerate receptor internalization, while inhibition of actin polymerization turns synapses in a permissive state. This modulatory role of synaptic activation in the expression and maintenance of synaptic plasticity raises important questions. Since the frequency of fEPSP recordings alters the time course of LTP and LTD expression and maintenance^11^,^30^ how do the temporal rules obtained using electrophysiological recordings translate to behavioural paradigms? Consistent with this role of synaptic activation in modulating the state of synapses, we found that suspending synaptic activation reduces the ability of activated synapses to capture PRPs, i.e. reduces the duration of the synaptic tag. The synaptic tag is time-restricted and it has been previously estimated to last around one hour^11^,^26^,^29^. Our results suggest that synaptic activation alters the time course of the synaptic-tag and that network-recurrent activity may be necessary to maintain synapses competent for protein capture during long periods of time. Depending on the experimental conditions, this may also explain the mismatch between the duration of the tag obtained using electrophysiological recordings, the time-course of phosphorylation of candidate molecules, such as CaMKII, and the time-course of actin dynamics using imaging approaches. It remains to be addressed whether a similar modulation of synaptic activation is observed in LTP synaptic-tagging and capture. If synaptic activation extends the duration of the tag by maintaining CaMKII active and actin filaments in a dynamic state, then upon learning the time-course of the network-recurrent activity will determine the duration of the synaptic tag and thus modulates the time-interval during which events can be associated.

## Materials and Methods

### Slice preparation

Slice preparation was performed as previously described^11^. In brief, male Sprague–Dawley rats (21–35 days old) were decapitated under isoflurane anesthesia; the brains were quickly removed and immersed in ice-cold artificial cutting cerebrospinal fluid (ACSF). In this study, we used 155 animals, from which 330 slices were used. Animals were housed in an Animal Facility at Instituto Gulbenkian de Ciência, licensed by Portuguese Veterinary Organization with the number PT 05 052 OICB. All procedures were conducted according to the regulations of the Portuguese Veterinary Organization (DGVA) and in accordance to the European regulations for the use of animals in experimental procedures (Regulation 86/609/CEE). All procedures were approved by DGVA (project license number 18831), The cutting ACSF was saturated with 95% O_2_/5% CO_2_ and contained (in mM) – NaCl, 126; KCl, 2.5; NaH_2_PO_4_, 1.25; NaHCO_3_, 26; MgCl_2_, 3; CaCl_2_, 2; glucose, 25. The hippocampi were isolated and cut into 400 μm-thick transverse slices using a vibratome (Leica, VT1200S). Slices were maintained in ACSF at 32 °C for at least 1 h before recording. They were then transferred to a submersion chamber and perfused continuously (1.5–2 mL/min) with recording ACSF at 32 °C. The recording ACSF was saturated with 95% O_2_/5% CO_2_ and contained (in mM) – NaCl, 126; KCl, 2.5; NaH_2_PO_4_, 1.25; NaHCO_3_, 26; MgCl_2_, 1; CaCl_2_, 2.5; glucose, 25.

### Electrophysiological recordings

Recordings started after a 20-min resting phase in the recording chamber. Schaffer collaterals were stimulated with 0.2 ms pulses using monopolar tungsten electrodes. Field excitatory postsynaptic potentials (fEPSP) were recorded extracellularly in the stratum radiatum of the CA1 region (approximately 130 μm below the slice surface) using glass microelectrodes filled with 3 mM NaCl immobilized with 1% agarose (tip resistance 3–5 MΩ). Stimulus intensities were set to evoke 50% of the maximal fEPSP slope. Test pulse frequency was 0.067 Hz. LTD was induced after recording a stable 40 min baseline of fEPSPs. To rule out unspecific effects of drugs or DMSO application on synaptic transmission, we performed I/O curves and PPF (pair-pulse facilitation) recordings. PPF was assessed by fEPSP responses evaluated at 10, 20, 40, 60, 120 ms inter-stimulus interval. Data points correspond to the average of three consecutive pulses for each value of inter-stimulus interval and averaged crossed experiments. I/O curves were obtained by fEPSP slope recordings elicited by increasing steps of 20 μA before and after drug application. Data points correspond to the average of three consecutive pulses for each value of injected current and averaged crossed experiments.

### Induction of synaptic plasticity

Two stimulating electrodes were positioned in the stratum radiatum layer, allowing the activation of two independent sets of Schaffer collaterals (please see inset in figures). Pathway independence was assessed by applying two pulses with 30 ms inter-pulse interval and confirming the absence of paired-pulse facilitation (PPF) between the pathways. After baseline stimulation, one of the pathways was arbitrarily chosen to receive strong low-frequency burst stimulation (S1-LFS - 3 pulses per burst at 20 Hz, 900 bursts repeated at 1 Hz). The second pathway served as a control pathway and was continuously recorded (S2). In the ‘synaptic capture’ experiments, weak long-term depression was induced by stimulation of one pathway (S2) with a weak low-frequency burst stimulation (W-LFS, 900 pulses at 1 Hz). After 30 min, strong long-term depression (L-LTD) was induced in a second independent pathway (S1) with an S-LFS. In a subset of experiments a third stimulation electrode was used to retrogradely stimulate the Schaffer collaterals and used as a control pathway (S3).

### Drug treatment

Drugs were dissolved in dimethylsulfoxide (DMSO - Sigma) and diluted down to achieve the final concentration – Cytochalasin-B (Sigma) 0.5 μM (in 0.01% DMSO); Rapamycin (Sigma) 1 μM (in 0.01% DMSO); Jasplakinolide (Invitrogen) 0.1 μM (in 0.01% DMSO); Kn-93 (Merck Chemicals) 1 μM (in 0.01% DMSO). For control experiments only DMSO (0.01%) was added to the ACSF.

### Data analysis

Electrophysiological data were collected using a Dagan IX2-700 amplifier (Dagan, Minnesota, USA) and band-pass filtered (low-pass 1 kHz, high-pass filter 1 Hz, LHBF 48X; NPI Electronic GmbH, Germany). Data were sampled at 5 kHz using a Lab-PCI-6014 data acquisition board (National Instruments, Austin, TX, USA) and stored on a PC. Offline data analysis was performed using a customized LabView-program (National Instruments). As a measure of synaptic strength, the initial slope of the evoked fEPSPs was calculated and expressed as percent changes from the baseline mean. Error bars denote SEM values. Experiments were rejected if the control pathways decayed more than 20% below baseline. After confirming homoscedasticity (Levene test) and normality (Kolmogorov-Smirnov test), group differences in LTD values between tested conditions were assessed at times T1 (40–50 min), T2 (70–80 min), T3 (100–110 min) and T4 (190–200 min). We have normalized LTD values for the percentage change, to baseline values, observed in the control pathway (S3). Group differences were assessed using repeated-measures ANOVA with a Tukey or Fisher *post hoc* test for multiple comparisons (Statistica StatSoft, DeLL software). No effect of time was observed within the time windows chosen. For input-output (I/O) curves and pair-pulse facilitation (PPF) analysis, a one-way ANOVA analysis was performed for each current or time-interval to assess group differences.

## Additional Information

**How to cite this article**: Szabó, E. C. *et al*. The interplay between neuronal activity and actin dynamics mimic the setting of an LTD synaptic tag. *Sci. Rep.* **6**, 33685; doi: 10.1038/srep33685 (2016).
